# Premenstrual Syndrome and Nutritional Factors: A Narrative Review of Current Evidence and Clinical Implications

**DOI:** 10.3390/jcm15031124

**Published:** 2026-01-31

**Authors:** Francesco Giuseppe Martire, Eugenia Costantini, Ilaria Ianes, Claudia d’Abate, Maria De Bonis, Emilio Piccione, Angela Andreoli

**Affiliations:** 1 Department of Molecular and Developmental Medicine, Obstetrics and Gynecological Clinic, University of Siena, 53100 Siena, Italy; francescogmartire@libero.it (F.G.M.); eugenia.costantini22@gmail.com (E.C.); ilaria.ianes@gmail.com (I.I.); claudiadabate94@gmail.com (C.d.); mariadebonis@gmail.com (M.D.B.); 2 Gynecology and Obstetrics, Department of Surgical Sciences, University of Rome “Tor Vergata”, 00133 Rome, Italy; 3 Residency Program of Gynecology and Obstetrics, Catholic University “Our Lady of Good Counsel”, 1000 Tirane, Albania; 4 Clinical Nutrition Unit, Department of System Medicine, Department of Surgical Sciences, University of Rome “Tor Vergata”, 00133 Rome, Italy; angela.andreoli@uniroma2.it

**Keywords:** complementary therapy, nutritional factor, premenstrual syndrome, prevention, symptoms

## Abstract

Premenstrual syndrome is a common hormone-related condition marked by recurrent physical and affective symptoms that can substantially impair daily functioning. While cyclical ovarian hormone fluctuations are physiological, clinically relevant symptoms occur only in a subset of women, indicating the contribution of individual vulnerability and modifiable environmental factors. In this context, growing attention has been directed toward the role of nutrition. This narrative review synthesizes and critically discusses current evidence on the relationship between dietary factors and premenstrual syndrome, with emphasis on both primary prevention and symptom modulation. Available observational and interventional data suggest that dietary patterns characterized by high intake of ultra-processed foods, refined carbohydrates, and saturated fats are more frequently associated with increased symptom severity, whereas healthier dietary patterns may be linked to a lower symptom burden. Certain micronutrients—including calcium, vitamin D, zinc, iron, and omega-3 fatty acids—have demonstrated potential benefits, although findings remain heterogeneous and methodologically limited. Overall, nutrition emerges as a plausible complementary strategy in premenstrual syndrome management; however, well-designed prospective studies are needed to support robust, evidence-based dietary recommendations.

## 1. Introduction

Premenstrual syndrome is a gynaecological and psychiatric disorder that reduces the quality of life of affected patients [1]. Depending on the severity of the somatic, behavioral and psychological symptoms, there are different degrees of the condition, ranging from premenstrual syndrome to premenstrual dysphoric disorder [2]. The pathogenesis of the disease is still unclear, but hormonal, genetic and environmental exposure factors certainly seem to play a key role in its onset in predisposed women [3]. Even with regard to the diagnostic definition of the various forms of pathology, there is no unanimous agreement, and different gynaecological and psychiatric societies use different symptoms to define the type and degree of disorder [4]. Epidemiological data provides interesting information that paves the way for nutrition to play a role in the development and treatment of this disorder. In fact, the incidence of the disease varies across continents and countries, which is why dietary habits may play an important role [5]. This consideration is very important because, in addition to traditional drug therapy, complementary nutritional therapy could play an important role in reducing the incidence of the disease from a primary prevention perspective and improving quality of life by reducing the disabilities caused by the disease when used as an adjunct therapy [6]. The aim of this narrative review is to evaluate the role of nutrition as a primary prevention strategy and to improve quality of life by reducing symptoms as a complementary therapy.

## 2. Materials and Methods

**Figure 1:**
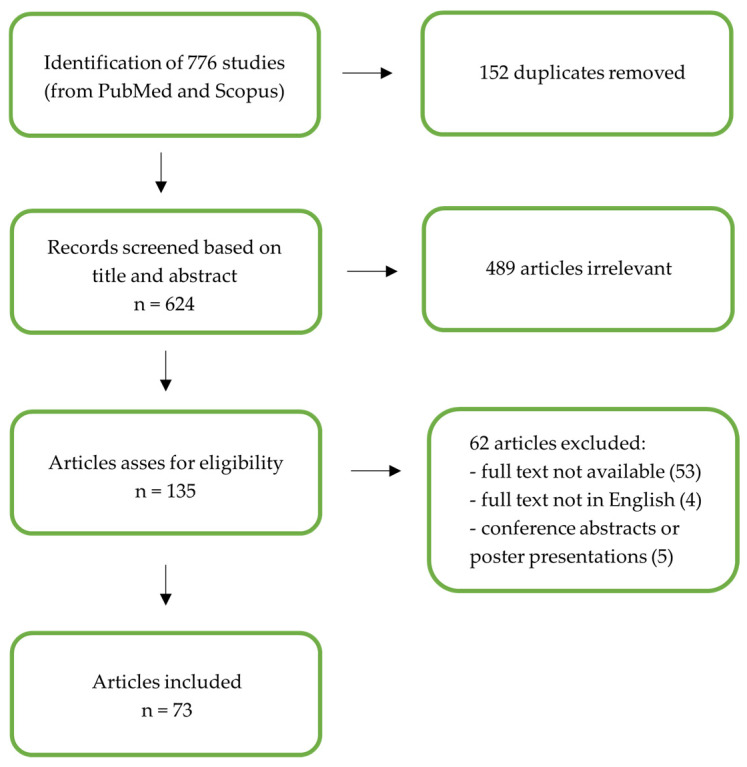
Inclusion criteria diagram.

An electronic literature search was conducted in MEDLINE (via PubMed) and Scopus to identify all English-language publications related to premenstrual disorder and nutritional factors from its inception through October 2025 (Figure 1). These databases were selected to ensure broad coverage of both biomedical and multidisciplinary literature relevant to premenstrual disorders and nutritional factors. PubMed was chosen as the primary biomedical database, while Scopus was included to capture additional journals and interdisciplinary sources not indexed in PubMed. Other databases were not searched in order to limit duplication and because the narrative design of the review did not require exhaustive systematic coverage. To retrieve relevant studies, we used combinations of specific keywords and Medical Subject Headings (MeSH), including: ‘Aliment’, ‘Diet’, ‘Nutrition’, ‘Nutritional Factors’, ‘Premenstrual Disorder’, ‘Premenstrual Dysphoric Disorder’, ‘Premenstrual Syndrome’.

Eligible sources comprised original investigations such as randomized and non-randomized clinical trials, prospective observational studies, retrospective cohort analyses, case–control studies, as well as review papers. Studies were included if they explicitly addressed the scope of this narrative review, namely, providing an overview of premenstrual syndrome in relation to nutritional factors.

Titles and abstracts were screened manually by two independent reviewers (F.G.M. and A.A.). Full-text articles were retrieved and assessed for eligibility using predefined inclusion criteria. Predefined exclusion criteria were applied during the screening and full-text assessment phases. Reasons for exclusion at the full-text stage are reported in the study selection process (Figure 1). No dedicated screening software was used, as the number of records was manageable and the narrative design of the review allowed for manual screening by two independent reviewers. This approach was considered appropriate to ensure accurate thematic assessment while maintaining transparency and reproducibility. Any discrepancies were resolved through discussion between the two reviewers and, when necessary, by consultation with the senior authors until full consensus was achieved.

The included studies were analyzed using a narrative thematic approach. Findings were grouped and synthesized into thematic areas reflecting epidemiology, pathophysiological mechanisms, clinical features, therapeutic approaches, and nutritional factors. A total of 776 studies were identified through searches of PubMed and Scopus. After the removal of 152 duplicate records, 624 articles underwent title and abstract screening, resulting in the exclusion of 489 studies deemed not relevant. The texts of 135 potentially eligible articles were then assessed, and 62 were excluded for the following reasons: 53 had no accessible full text, 4 were not published in English, and 5 were conference abstracts or poster presentations. Ultimately, 73 studies met the inclusion criteria and were included in the review (Figure 1).

## 3. Results

The results of the narrative synthesis are presented below according to the predefined thematic areas.

### 3.1. Epidemiology

Premenstrual syndrome (PMS) is characterized by recurrent physical and psychological symptoms that emerge during the luteal phase of the menstrual cycle and typically resolve with the onset of menstruation. Globally, the estimated prevalence of PMS is 47.8%, while premenstrual dysphoric disorder (PMDD) affects approximately 3–8% of reproductive-aged women [5]. The reported prevalence of premenstrual disorders varies widely across studies and populations. Longitudinal data show that symptom severity and persistence can fluctuate substantially over time: in one study, only 36% of women who initially met diagnostic criteria for PMS still fulfilled them one year later, indicating considerable instability in symptom expression [7]. PMDD, the more severe form of the disorder, affects a smaller proportion of women, with estimates ranging from 1.3% to 5.3% [2]. Substantial geographical variability has been documented. In Japanese women attending a gynecologic cancer screening clinic, the prevalence of moderate to severe PMS and PMDD was 5.3% and 1.2%, respectively [8]. In Switzerland, a nationwide survey showed that 91% of women reported at least one premenstrual symptom, with 10.3% meeting the diagnostic criteria for PMS and 3.1% for PMDD [9]. Chinese population-based study of women aged 18–45 years, PMS prevalence reached 21.1%, while PMDD was reported in 2.1% of participants [10]. A recent meta-analysis estimated the global prevalence of PMS at 47.8%, with reported values ranging from 12% in France to 98% in Iran [5]. In Africa, a 2024 systematic review reported a pooled PMS prevalence of 46.98%, further underscoring the marked regional differences observed across populations [11]. The striking inter-country variability is influenced not only by true differences in symptom distribution but also by substantial methodological heterogeneity across studies. Variations in diagnostic criteria (e.g., prospective daily ratings vs. retrospective questionnaires), sample selection, symptom severity thresholds, and cultural norms regarding the perception and reporting of distress greatly affect prevalence estimates. Many studies also rely on small, non-representative samples or use differing definitions of “moderate” and “severe” PMS, limiting direct comparability. Furthermore, sociocultural factors—including attitudes toward menstruation, stigma, stress exposure, and lifestyle patterns—likely shape both symptom expression and help-seeking behaviors. Substantial methodological heterogeneity was observed across studies, including differences in diagnostic criteria, sample selection, symptom severity thresholds, and cultural contexts. Several biological and lifestyle-related factors have been explored as potential contributors to PMS susceptibility. Some evidence suggests that women with Rh-negative blood type may have a higher likelihood of developing premenstrual symptoms, although mechanistic explanations remain speculative and findings are not yet consistent across studies. High caffeine intake has also been linked to increased PMS risk, possibly through its impact on sleep quality, anxiety, and neuroendocrine regulation. Additionally, younger age at menarche has been identified as a potential vulnerability factor, with the hypothesis that earlier initiation of cyclical ovarian hormone exposure may predispose to dysregulated neuroendocrine responses later in reproductive life [12]. While none of these factors is sufficient to cause PMS on its own, they may contribute to a broader vulnerability profile interacting with genetic predisposition, hormonal sensitivity, and psychosocial stressors.

### 3.2. Pathogenesis

The etiopathogenesis of PMS and PMDD is multifactorial and remains only partially understood, but current evidence indicates that symptoms arise from a complex interplay between normal ovarian steroid fluctuations and an underlying neurobiological vulnerability present only in susceptible individuals. Although estrogen and progesterone levels do not differ substantially between symptomatic and asymptomatic women, the central nervous system of affected individuals appears to respond abnormally to these physiological variations. Experimental studies using GnRH agonists, which suppress ovarian activity, have shown that women with PMDD develop mood symptoms only after estradiol and progesterone reintroduction. In contrast, healthy controls do not exhibit comparable changes. These findings support altered sensitivity to hormonal fluctuations rather than a quantitative hormonal abnormality [13]. A key mechanism involves neurosteroids derived from progesterone, particularly allopregnanolone, a potent modulator of the GABA A receptor. In PMS and PMDD, GABA A receptor plasticity is dysregulated, resulting in paradoxical or blunted responses to allopregnanolone and to pharmacological agents acting on the same receptor complex. This dysfunctional neurosteroid–GABAergic adaptation contributes to emotional lability and heightened stress reactivity during the luteal phase, and interacts with other systems such as serotonergic signaling and neural stress circuits [3]. Serotonergic dysregulation represents another critical component: variations in serotonin sensitivity and metabolism across the menstrual cycle may predispose susceptible women to mood instability, consistent with the established efficacy of SSRIs in these disorders [14]. Increasing attention has been directed toward the role of immune and stress-responsive systems. Although the evidence on inflammation remains heterogeneous, several studies suggest that low-grade inflammatory activity and oxidative stress, partially modulated by ovarian steroid fluctuations, may exacerbate premenstrual symptoms [15,16,17]. The hypothalamic–pituitary–adrenal (HPA) axis, a major regulator of stress responses, also appears to be altered. Women with PMS show abnormal cortisol reactivity to stressors—either blunted or exaggerated—patterns that differ from those observed in asymptomatic individuals and may contribute to mood and somatic symptoms [18,19,20]. Stress itself has emerged as a major vulnerability factor. A large body of research indicates that women with PMS or PMDD exhibit heightened subjective stress, increased emotional reactivity, and greater symptom exacerbation in response to acute and chronic stressors. A systematic review reported that stress was identified as a significant contributor to PMS symptomatology in 92% of included studies, while women with PMDD more frequently reported histories of trauma or major life stress [13]. Activation of stress pathways—including the HPA axis and noradrenergic centers—can suppress GnRH release, alter LH and FSH secretion, and modulate ovarian steroid production, potentially worsening luteal-phase vulnerability [21]. These stress-related pathways interact with immune responses: inflammatory cytokines can influence neurotransmission, alter GABAergic and serotonergic function, and contribute to central sensitization and mood dysregulation [22]. Emerging evidence also implicates the orexin (hypocretin) system, which regulates arousal, mood, cognition, neuroendocrine signaling, and inflammatory responses—domains frequently affected in PMS. Altered orexinergic tone may contribute to sleep disturbances, affective instability, cognitive complaints, and heightened stress sensitivity during the luteal phase. Orexin interacts with ovarian steroids, exerts anti-inflammatory actions, and modulates GABAergic transmission, thereby linking multiple biological pathways relevant to PMS [23]. Genetic and individual vulnerability factors further modulate these biological systems. Twin and family studies indicate heritability of premenstrual disorders, although specific genetic variants have not yet been identified [13]. Structural and functional changes in neural circuits involved in emotional regulation have been documented, suggesting that neurocircuitry differences condition susceptibility to hormonal and stress-related triggers. Environmental and lifestyle factors—including diet, micronutrient status, caffeine and alcohol intake, smoking, and chronic stress—may also influence symptom expression. Dietary patterns and nutrient deficiencies appear to interact with neuroendocrine and metabolic pathways, modulating mood, inflammation, and energy regulation across the cycle [13]. The reviewed studies consistently described interactions between ovarian steroid fluctuations, neurotransmitter systems, stress reactivity, and immune signaling in women with PMS and PMDD. PMS and PMDD therefore represent cyclical disorders of neuroendocrine and neurobiological sensitivity, rather than conditions driven by isolated endocrine abnormalities [3,22].

### 3.3. Symptoms and Diagnosis

Premenstrual syndrome is a clinical condition characterized by the recurrent occurrence of physical, psychological, and behavioral symptoms during the luteal phase of the menstrual cycle, with spontaneous resolution at the onset of menstruation or shortly thereafter. Its clinical presentation is highly heterogeneous: more than 200 different symptoms have been described, occurring with variable intensity and in diverse combinations. The most frequently reported manifestations include abdominal bloating, breast tenderness, headache, fatigue, myalgia, appetite changes, irritability, emotional lability, anxiety, depressed mood, and impaired concentration. In a substantial proportion of affected women, these symptoms lead to a meaningful reduction in quality of life and impair social, occupational, and academic functioning [1,24,25].

The main symptom domains associated with premenstrual syndrome are summarized in Table 1.

From a diagnostic perspective, the defining feature of PMS is not the specificity of individual symptoms but rather their cyclical pattern and temporal association with the menstrual cycle. The American College of Obstetricians and Gynecologists (ACOG) defines PMS as the presence of at least one affective or somatic symptom of sufficient severity to interfere with daily activities, provided that symptoms are documented for at least two consecutive cycles [4]. Premenstrual dysphoric disorder represents the most severe end of the spectrum and is defined in the Diagnostic and Statistical Manual of Mental Disorders, Fifth Edition, Text Revision (DSM-5-TR) by more stringent criteria, requiring at least five symptoms—predominantly affective—associated with marked functional impairment [26]. A clinically important issue is the differential diagnosis between PMS/PMDD and premenstrual exacerbation (PME), which refers to the worsening of pre-existing psychiatric or medical conditions during the premenstrual phase. In PME, symptoms are not confined to the luteal phase but persist, albeit less intensely, throughout the cycle. To improve diagnostic accuracy, current guidelines recommend prospective daily symptom rating for at least two menstrual cycles. Nevertheless, recent studies indicate that such prospective tools are infrequently used in routine clinical practice, contributing to a substantial gap between formal diagnostic criteria and real-world diagnoses [27]. This is particularly relevant in adolescents, in whom cyclical pelvic pain and overlapping premenstrual symptomatology may delay recognition of underlying gynecological conditions and complicate clinical management [28]. Within this complex clinical and diagnostic framework, increasing attention has been directed toward potentially modifiable factors influencing symptom severity and expression. Among these, dietary habits and nutritional status are gaining relevance as possible determinants of premenstrual vulnerability. A similar paradigm is already emerging in other chronic gynecological conditions, such as endometriosis, where targeted dietary interventions appear to modulate inflammation, pain perception, and quality of life, acting as complementary strategies alongside conventional pharmacological treatment [29,30,31]. This evolving evidence supports the hypothesis that nutrition may also represent a meaningful component of both primary prevention and integrated clinical management in PMS.

### 3.4. Classification

The classification of premenstrual conditions has evolved over time, reflecting an improved understanding of their clinical heterogeneity. Traditionally, PMS has been used in gynecological settings to describe cyclic physical and psychological symptoms, whereas PMDD was introduced in psychiatric nosology to identify a more severe form dominated by affective symptoms and marked functional impairment. More recently, the umbrella concept of Premenstrual Disorders (PMDs) has been proposed, framing PMS and PMDD as points along a continuum of severity rather than as discrete entities [27,32]. Within this framework, the International Society for Premenstrual Disorders (ISPMD) has proposed a classification distinguishing core PMDs, which include PMS and PMDD, from variant PMDs, such as premenstrual exacerbation of pre-existing medical or psychiatric disorders. This approach emphasizes functional impairment and symptom cyclicity rather than rigid symptom counts, making it particularly applicable to clinical practice. Indeed, many women experience clinically significant and disabling symptoms without fulfilling the full DSM criteria for PMDD, yet still require therapeutic intervention. The coexistence of multiple classification systems—including ACOG, DSM, ICD, and ISPMD—represents a major source of heterogeneity in the scientific literature. This variability has important epidemiological and methodological implications, influencing prevalence estimates, population selection, and interpretation of findings, particularly in studies exploring nutritional factors. The absence of a universally accepted classification hampers direct comparison across studies and complicates the identification of patient subgroups that may benefit most from targeted dietary interventions.

The main classification systems for premenstrual conditions are outlined in Table 2.

### 3.5. Comorbidity

When comorbid conditions are present, patients may experience a worsening of symptoms during the premenstrual phase. For example, many women with IBD report cyclical fluctuations in gastrointestinal complaints, and more than 70% describe a perimenstrual exacerbation of their symptoms [33].

Variations in gastrointestinal (GI) function across the menstrual cycle are thought to reflect the presence of sex hormone receptors throughout the GI tract [33].

Although such fluctuations are also observed in healthy women, they tend to be more pronounced in those with inflammatory bowel disease (IBD) and in individuals with other abdominal inflammatory conditions, including familial Mediterranean fever (FMF) and Behçet’s disease [34,35,36]. Moreover, endometriosis—particularly deep infiltrating disease—has been reported with increased frequency in women with IBD, reinforcing the need for careful differential diagnosis when pelvic pain and gastrointestinal symptoms coexist [37].

The precise mechanisms driving menstrual-related symptom worsening remain uncertain. One proposed explanation involves prostaglandins (PGs). During menstruation, uterine synthesis of PGs—particularly PGF_2_α and prostacyclin—increases substantially, and these mediators are known to stimulate intestinal motor activity [33].

Given their established role in inflammatory pathways relevant to IBD, the heightened PG release from the endometrium during menses may further intensify gastrointestinal symptoms.

Estrogenic signaling represents an additional pathway potentially contributing to these fluctuations. Recent findings indicate a broad distribution of estrogen receptors along the GI tract. Three subtypes have been identified: estrogen receptor α (ERα), estrogen receptor β (ERβ), and the G protein–coupled estrogen receptor (GPER) [38]. Although ERα and ERβ are expressed throughout the upper GI tract, ERβ predominates in the colon, where it supports epithelial integrity and provides protection against chronic colitis [39,40,41,42]. Hormonal influences on visceral sensitivity and motility are further supported by evidence from irritable bowel syndrome (IBS), where estrogen-dependent mechanisms—particularly those mediated through GPER—have been implicated in modulating pain perception and GI transit [43,44].

These observations suggest that cyclical changes in estrogen levels may meaningfully affect both intestinal inflammation and motility in women with IBD.

Although the literature has extensively documented an exacerbation of intestinal symptoms around menstruation in this patient population [34,35,36,45,46], data aimed at identifying specific risk factors for menstrual cycle–associated clinical worsening remain scarce.

A bidirectional association between migraine and endometriosis has been increasingly reported, suggesting shared mechanisms related to neuroinflammation, central sensitization, and hormone-dependent modulation of pain processing. These links may contribute to cyclical exacerbations and a higher overall symptom burden in susceptible individuals [47].

### 3.6. Medical Therapy

The management of premenstrual syndrome is primarily aimed at reducing symptom burden and improving quality of life. A crucial step is the identification of predominant symptoms—whether physical, psychological, or behavioral—in order to guide the selection of the most appropriate therapeutic strategy and achieve optimal clinical benefit for each patient.

A comprehensive, multidisciplinary approach involving the general practitioner, gynecologist, and psychiatrist, with the possible contribution of other healthcare professionals such as nutritionists, is particularly valuable for holistic assessment and individualized management of PMS, including the use of complementary therapeutic strategies.

Historically, hormonal therapy has represented a cornerstone in the treatment of PMS, with the objective of minimizing or abolishing cyclical fluctuations in sex hormone levels. This strategy may attenuate adaptive changes within the central nervous system that are driven by progesterone, its metabolites, and estrogens.

In this context, the absence of the progestogenic peak may prevent maladaptive responses of GABA-A receptors to allopregnanolone [48,49]. Suppression of this mechanism could also contribute to increased serotonin levels in women affected by PMS [1].

Based on these neuroendocrine interactions, monophasic combined oral contraceptives (COCs) appear to represent a more suitable therapeutic option than multiphasic formulations. The latter are characterized by a progressive increase in the progestin component during the second half of the cycle, thereby mimicking physiological sex hormone fluctuations. In contrast, monophasic preparations eliminate the progesterone peak, reducing the likelihood of dysfunctional neurobiological adaptation [3].

Accordingly, current clinical guidelines recommend monophasic formulations for the management of mood-related symptoms associated with premenstrual syndrome [50].

#### 3.6.1. Hormonal Treatment

The clinical efficacy of combined oral contraceptives (COCs) in the management of premenstrual disorders is mediated by several complementary mechanisms. A key component of their action is the suppression of ovulation, achieved through stabilization of circulating sex hormone levels by both hormonal components. This stabilization may also contribute to improved mood regulation. Among the available formulations, those containing ethinylestradiol and drospirenone appear to confer the greatest clinical benefit and are approved by the U.S. Food and Drug Administration for the treatment of premenstrual dysphoric disorder [3]. In addition to ovulation suppression, these formulations exert antiandrogenic effects that may help alleviate symptoms such as irritability and aggression.

Evidence suggests that androgens may play a role in premenstrual symptomatology. Eriksson et al. reported elevated serum testosterone concentrations in women with premenstrual symptoms, irrespective of the menstrual cycle phase [51], supporting a potential contribution of androgenic activity in susceptible individuals.

Drospirenone is a synthetic progestogen derived from progesterone and exhibits antiandrogenic activity up to ten times greater than that of the parent hormone. Its beneficial effects in PMS and PMDD are thought to be mediated, at least in part, through antagonism of the mineralocorticoid receptor [52]. Structurally and pharmacologically, drospirenone is an analogue of spironolactone, a compound known for both its diuretic properties and its positive effects on mood [49].

The mood improvement observed with spironolactone may be related to its ability to reduce and normalize circulating progesterone levels [3], there by potentially preventing maladaptive neurobiological responses to progesterone and its neuroactive metabolite allopregnanolone. This mechanism provides a plausible explanation for the favorable neuropsychological profile associated with drospirenone-containing formulations.

Initial clinical trials of drospirenone-based contraceptives produced inconsistent results. The relatively long hormone-free interval used in early regimens (21/7 days) may partly account for these findings, as improvements were largely limited to somatic symptoms such as acne, appetite changes, and breast tenderness, with minimal effects on mood. In contrast, studies employing shorter placebo intervals (24/4 days) demonstrated significant improvements in both physical symptoms—including mastalgia, bloating, abdominal distension, headache, and myalgia—and mood. Nevertheless, previous investigations have emphasized that, although COCs are effective in alleviating physical symptoms, selective serotonin reuptake inhibitors (SSRIs) remain more effective for mood symptoms in PMDD [53].

As with other pharmacological treatments, drospirenone-containing COCs may be associated with adverse effects, most commonly nausea, breast tenderness, and intermenstrual bleeding [54].

In contrast, progestogen-only oral contraceptives are not recommended for the treatment of PMS and PMDD, as they may exacerbate mood instability and other premenstrual symptoms [55]. Consistent with this observation, women with mood disorders have been shown to exhibit higher circulating progesterone levels compared with healthy controls [56].

Estrogen-only therapy is similarly discouraged in this clinical context. Available evidence indicates that estrogen monotherapy is either ineffective or may worsen premenstrual symptoms [57]. Moreover, unopposed estrogen exposure is associated with an increased risk of endometrial cancer. Consequently, combined hormonal preparations represent a safer and more effective therapeutic strategy, as previously discussed [58].

Another important consideration is the hormone-free interval. Despite their overall efficacy, combined oral contraceptive regimens that include a placebo phase may fail to fully suppress cyclical hormonal fluctuations [59].

Continuous combined oral contraception may suppress oscillations in luteinizing hormone (LH), follicle-stimulating hormone (FSH), estradiol, and progesterone, thereby further improving symptom control and overall well-being [3].

In support of this approach, Halbreich et al. (2012) evaluated a formulation containing levonorgestrel (LNG) 90 μg and ethinylestradiol 20 μg administered over four consecutive 28-day cycles [59]. More than half of the treated women achieved a clinically meaningful response, defined as a ≥50% reduction in symptom severity, with higher response rates observed with longer treatment duration [59].

Beyond symptom relief, COC therapy has also been shown to improve bleeding control and reduce dysmenorrhea [3]. Accordingly, these agents appear particularly beneficial in women with more severe forms of PMS and PMDD, especially when physical symptoms are prominent and insufficiently controlled.

Finally, it should be noted that levonorgestrel-releasing intrauterine devices (LNG-IUDs) may increase sensitivity to stress. Women using LNG-IUDs have been reported to exhibit significantly higher cortisol levels than those receiving oral levonorgestrel in combination with estrogens, potentially reflecting increased autonomic nervous system reactivity to stressors [60]. Several studies have also suggested an association between LNG-IUD use and worsening of mood symptoms [60,61,62].

In summary, among contraceptive-based interventions, combined hormonal therapy—particularly drospirenone-containing formulations administered in a continuous regimen—appears to offer the most consistent benefit for the management of symptoms associated with premenstrual syndrome and PMDD.

#### 3.6.2. GnRH Agonist and Antagonist Treatment

These agents also play an important role in the management of premenstrual syndrome and premenstrual dysphoric disorder. Their mechanism of action involves suppression of the hypothalamic–pituitary–ovarian axis, resulting in inhibition of ovulation. However, they should be regarded as second-line therapies and used for limited durations, given that they induce a state of pharmacological menopause associated with adverse effects such as bone mineral density loss and vasomotor symptoms [3].

To mitigate menopause-like adverse effects related to GnRH analogue therapy, hormonal “add-back” regimens have been proposed. At present, the number of studies evaluating the efficacy of GnRH agonists in combination with add-back therapy remains limited, making it difficult to draw definitive conclusions. A meta-analysis by Wyatt et al. suggested that add-back therapy does not reduce the therapeutic efficacy of GnRH agonists [63].

Further studies in women with PMS and PMDD are therefore needed to confirm these findings and to define the role of this therapeutic approach in carefully selected patient subgroups.

GnRH antagonists rapidly suppress pituitary gonadotropin secretion through competitive inhibition of GnRH receptors, thereby avoiding the initial stimulatory phase characteristic of GnRH agonists [3].

The potential utility of GnRH antagonists in the treatment of PMS and PMDD lies in their rapid onset of action and the prompt recovery of pituitary function following treatment discontinuation [64]. Unlike agonists, which require prolonged and continuous administration to maintain efficacy, antagonists offer a more predictable pharmacological profile. However, their use is limited by high cost and the possible need for add-back hormone therapy [65].

#### 3.6.3. Antidepressant Medication

Selective serotonin reuptake inhibitors (SSRIs) are widely recognized as an effective pharmacological option for the management of severe premenstrual syndrome and premenstrual dysphoric disorder. International guidelines, including those from the Royal College of Obstetricians and Gynaecologists, identify SSRIs as first-line therapy for severe forms of these conditions, particularly for the treatment of affective symptoms such as irritability, anxiety, and mood instability [66,67].

Clinical evidence indicates that SSRI treatment leads to clinically meaningful symptom improvement in a substantial proportion of women with PMDD when compared with placebo [68]. Adverse effects are generally consistent with the known safety profile of this drug class and most commonly include gastrointestinal symptoms, fatigue, and sexual dysfunction [1,69].

A distinctive feature of SSRIs in PMS and PMDD is their rapid onset of therapeutic action, which allows for either continuous administration or intermittent dosing limited to the luteal phase of the menstrual cycle [55,69]. Available studies have not demonstrated a clear superiority of one dosing regimen over the other, and treatment choice may therefore be guided by patient preference, tolerability, and clinical judgment [69,70].

Overall, SSRIs represent an established and effective therapeutic option for severe PMS and PMDD, with careful individualization of treatment being essential to optimize clinical outcomes while minimizing adverse effects [69].

#### 3.6.4. Cognitive Behavioural Therapy

Cognitive behavioural therapy (CBT) is a form of psychotherapy aimed at identifying and modifying negative cognitive experiences and maladaptive thought patterns in order to promote behavioural change. CBT has been shown to be an effective approach when combined with SSRI therapy. While SSRIs are associated with a more rapid onset of clinical benefit, CBT appears to provide more sustained symptom improvement when applied consistently over time [71].

#### 3.6.5. Physical Activity

Regular physical activity increases β-endorphin levels, contributes to the regulation of sex steroid synthesis, and promotes the production of endogenous compounds with anti-inflammatory properties [71].

### 3.7. Nutritional Factor

In order to alleviate the pain-related symptoms associated with PMS, several non-pharmacological strategies have been proposed, including targeted modifications of dietary intake. However, the currently available evidence regarding the association between diet and PMS remains heterogeneous and, in some cases, conflicting. Most studies focused on individual nutrients or dietary components rather than overall dietary patterns. Moreover, PMS itself may influence dietary choices, which in turn can contribute either to the exacerbation or to the improvement of symptoms. Consequently, although existing data suggest an association between dietary composition and PMS, they do not allow for the establishment of a clear causal relationship. This bidirectional relationship highlights an important gap in current research, namely the lack of longitudinal and mechanistic studies capable of disentangling cause–effect pathways. Nevertheless, increasing scientific attention is being devoted to the potential role of dietary patterns in other gynecological conditions, including endometriosis and uterine fibroids. This broader interest reflects the chronic inflammatory and hormone-dependent background shared by several benign gynecological disorders, such as endometriosis and adenomyosis [72]. In line with this perspective, recent evidence suggests that adenomyosis is associated with alterations in ovarian reserve markers, such as anti-Müllerian hormone (AMH), although the relationship remains complex and not fully elucidated, further supporting the concept of shared endocrine and inflammatory pathways across benign gynecological conditions [73]. In this context, PMS may be conceptualized within a wider spectrum of gynecological conditions in which diet could modulate inflammation, steroid hormone metabolism, and neurotransmitter synthesis. While the evidence remains preliminary, these emerging findings suggest that diet may represent a relevant, albeit not yet fully defined, modifiable factor across a broader spectrum of gynecological disorders [29,31].

#### 3.7.1. Macronutrients

Within this context, Houghton et al. [74] reported no association between fiber, carbohydrate, or protein intake and PMS. In contrast, other studies have documented a significant increase in premenstrual consumption of fats and simple carbohydrates, accompanied by a reduction in protein intake among women with PMS [75]. Such dietary shifts may have biological relevance, as macronutrient composition is known to influence central nervous system function and the availability of neurotransmitter precursors, including tryptophan for serotonin synthesis.

A significant inverse association has also been observed between PMS severity and the consumption of fish and seafood [76]. This finding may be partially explained by the anti-inflammatory properties of omega-3 fatty acids and their potential role in modulating serotonergic and endorphinergic pathways involved in mood and pain perception. Conversely, an increased risk of developing PMS symptoms has been linked to dietary patterns characterized by high intakes of red and processed meat, fast food, vegetable oils, mayonnaise, fried foods, salty snacks, refined grains, sugars and sugar-sweetened beverages, high-fat dairy products, spices, and fried potatoes [77]. Additional evidence indicates a positive association between the presence and severity of PMS symptoms and diets rich in simple carbohydrates, fried foods, and alcohol, alongside an inverse association with dietary patterns characterized by higher intakes of vegetables, fruits, and fiber [74,77,78,79,80]. The reviewed studies reported associations between dietary patterns characterized by higher consumption of fruits, vegetables, and fish and lower PMS symptom severity, whereas Western-style dietary patterns were more frequently associated with worse symptom profiles.

Consequently, white meat and fish—characterized by a lower saturated fat content and, in the case of fish, by the provision of omega-3 fatty acids—could represent nutritionally more favorable options within complementary dietary strategies.

In particular, fruit consumption has been associated with a reduced risk of psychological symptoms of PMS [81].

Other studies have failed to identify significant associations between fiber or carbohydrate intake and the development of PMS, with the exception of maltose. A higher intake of this disaccharide has been associated with a 45% increased risk of PMS, even after adjustment for body mass index, smoking, and other potential confounding factors [82].

A large cohort study found no association between total fat intake and the risk of PMS [83]. In contrast, a higher intake of stearic acid was associated with a reduced risk of developing PMS [82]. Similarly, no significant association has been observed between protein intake and PMS [84]. Importantly, the absence of consistent associations may reflect methodological heterogeneity, including differences in dietary assessment tools, PMS diagnostic criteria, and the lack of evaluation of diet-induced neurotransmitter regulation.

The main findings regarding the association between macronutrient intake and PMS are summarized in Table 3.

Overall, evaluating the relationship between PMS onset, symptom severity, and macronutrient intake remains particularly challenging, as reflected by the substantial inconsistencies reported in the literature. Consequently, based on the evidence currently available, it is not possible to formulate specific dietary recommendations aimed at reducing the severity of PMS symptoms. Future research should move beyond single-nutrient approaches and focus on integrative dietary models capable of supporting neurotransmitter homeostasis (e.g., serotonin and β-endorphin) and addressing the current gaps in mechanistic understanding.

#### 3.7.2. Micronutrients and Vitamins

Several hypotheses have been proposed to clarify the possible relationship between micronutrient and vitamin deficiencies and the development of premenstrual syndrome. In particular, some studies suggest that PMS may represent a clinical manifestation of calcium deficiency. This hypothesis is supported both by fluctuations in serum calcium levels during the menstrual cycle and by the relationship between calcium homeostasis and affective disorders [84]. Moreover, women affected by PMS have been shown to have a lower dietary intake of calcium, magnesium, and potassium compared with asymptomatic women [84]. These observations suggest that alterations in mineral balance may influence not only physical symptoms but also the neuropsychiatric regulation associated with PMS. Supporting these observations, supplementation with 1200 mg/day of calcium carbonate for three menstrual cycles in women with premenstrual dysphoric disorder resulted in a 48% reduction in the severity of both psychological and physical symptoms [85].

Further evidence indicates an association between vitamin D insufficiency and an increased risk of developing PMS [86], as well as greater symptom severity [87]. However, dietary vitamin D intake does not appear to influence the risk of PMS onset [88,89]. This discrepancy highlights a potential difference between dietary intake and systemic vitamin status, suggesting that factors such as sun exposure, absorption, and metabolism may play a decisive role. Conversely, supplementation with 50,000 IU/week of vitamin D has been shown to reduce the incidence of several PMS symptoms, including low back pain and crying tendency, as well as to decrease the severity of dysmenorrhea in adolescents [90]. Similarly, a supplementation regimen consisting of an initial dose of 200,000 IU followed by 25,000 IU every two weeks for four months improved mood-related PMS symptoms in young women with severe vitamin D deficiency [91]. The effectiveness of vitamin D supplementation in reducing PMS symptom severity has also been confirmed by other studies [92,93,94].

Another hypothesis implicates iron deficiency, as a high iron intake—particularly non-heme iron—has been associated with a significant reduction in the risk of developing PMS [95]. Recent evidence indicates that iron status may influence specific premenstrual symptoms rather than the syndrome as a whole. In particular, a genetically increased risk of iron overload has been associated with a lower likelihood of experiencing cognitive and somatic symptoms, such as confusion, headache, and nausea, whereas no significant associations have been observed with low iron status [94].

This effect may be mediated by the role of iron in neurotransmitter synthesis and cerebral energy metabolism, as well as by estrogen-dependent regulation of the hepcidin–ferroportin axis d during the luteal phase of the menstrual cycle, although the underlying mechanisms remain to be clarified.

With regard to zinc, a randomized clinical trial demonstrated that supplementation with 220 mg/day of elemental zinc for 24 weeks significantly reduced PMS symptom severity and improved quality of life compared with placebo [96,97]. In contrast, supplementation with thiamine and riboflavin has not been shown to significantly reduce PMS symptom severity [84,98]. These contrasting results underline the need to distinguish between micronutrients with direct biological effects on neurotransmission and those with a more limited role in PMS pathophysiology.

Studies evaluating the effectiveness of vitamin B6 have yielded inconsistent results. In one study, no significant differences in symptom severity were observed between women treated with vitamin B6 (80 mg/day) and those receiving broad-spectrum micronutrient supplementation for three menstrual cycles; however, complete symptom remission was observed in 72% of women treated with micronutrients and in 60% of those treated with vitamin B6 [84]. Conversely, a meta-analysis of 12 case–control studies including 586 women with PMS treated with vitamin B6 and 602 receiving placebo reported significant improvements in both physical and psychological symptoms in the vitamin B6 group [99]. These discrepancies may reflect differences in dosage, treatment duration, and diagnostic criteria adopted across studies.

Several trials have also demonstrated that supplementation with 80 mg/day of thiamine for two menstrual cycles is associated with a significant reduction in PMS symptom severity compared with placebo [84,100]. However, clinical trials evaluating vitamin B1 and calcium supplementation also highlight the absence of a standardized therapeutic approach for PMS. Although supplementation has consistently been shown to reduce symptom severity, marked interindividual variability and the presence of multiple confounding factors limit the translation of these findings into structured or universally applicable dietary recommendations [99].

A meta-analysis of eight randomized controlled trials further indicated that omega-3 fatty acids may contribute to reducing PMS symptom severity, although their effectiveness appears to depend on the duration of supplementation [101]. Omega-3 fatty acids exert anti-inflammatory effects through competitive mechanisms with arachidonic acid as substrates for cyclooxygenases and 5-lipoxygenases. In particular, eicosapentaenoic acid and docosahexaenoic acid reduce inflammatory processes by inhibiting leukocyte chemotaxis, modulating the expression of adhesion molecules and leukocyte–endothelium interactions, suppressing eicosanoid production, and reducing the synthesis of pro-inflammatory cytokines [84]. However, the optimal dosage and minimum duration of treatment required to achieve clinically meaningful benefits remain to be defined. Consequently, omega-3 fatty acid intake may help alleviate inflammation-related PMS symptoms.

The association between caffeine consumption and PMS has also been investigated, yielding conflicting results. Some studies have reported a strong positive association between caffeine intake and PMS symptom severity [102,103], whereas others have not confirmed these findings [104,105]. These inconsistencies suggest a possible dose-dependent effect or individual susceptibility that has not been adequately addressed in available studies.

Finally, a recent study showed that treatment with *Vitex agnus-castus* for approximately three months was associated with a marked improvement in symptoms—particularly dysmenorrhea and mastodynia—and quality of life in women with premenstrual syndrome [106]. Despite these promising findings, further studies are needed to clarify the mechanisms of action and to compare its effectiveness with other nutritional strategies.

In conclusion, various nutritional strategies can be fully included among complementary treatments; however, further studies—particularly randomized controlled trials—are required to more precisely define their efficacy, safety, and optimal modalities of use. In particular, future research should adopt an integrative approach aimed at addressing existing gaps and clarifying how micronutrients and vitamins may modulate the neuroendocrine and inflammatory mechanisms involved in PMS.

An overview of the evidence on micronutrients, vitamins, and complementary dietary treatments in PMS is provided in Table 4.

## 4. Discussion

### 4.1. Main Finding

This narrative review highlights the multifactorial nature of premenstrual syndrome, in which neuroendocrine sensitivity to physiological hormonal fluctuations interacts with individual vulnerability and lifestyle-related factors. The thematic results suggest that epidemiological variability across populations may reflect, at least in part, differences in environmental exposures, including dietary habits, although methodological heterogeneity and cultural factors substantially limit cross-study comparisons. Current evidence supports the concept that premenstrual syndrome and premenstrual dysphoric disorder arise from altered central nervous system responsiveness to normal ovarian steroid changes rather than from abnormal hormone levels. Neurosteroid–GABAergic mechanisms, serotonergic pathways, stress reactivity, and immune signaling appear to converge in susceptible individuals, contributing to cyclical symptom expression. Taken together, the available evidence supports an integrated etiological model in which ovarian steroid fluctuations serve as physiological triggers, but symptoms emerge only when these fluctuations interact with pre-existing vulnerabilities in neurotransmitter systems, stress regulation, immune pathways, and neural circuitry. Within this framework, nutritional factors emerge as a potentially relevant but non-causal component. Dietary patterns characterized by high intake of ultra-processed foods, refined carbohydrates, and saturated fats are more frequently associated with greater symptom burden, whereas healthier dietary patterns appear linked to more favorable profiles. Several micronutrients, including calcium, vitamin D, zinc, iron, and omega-3 fatty acids, have shown potential benefits; however, findings remain heterogeneous and largely based on observational data. Overall, these data support the hypothesis that overall dietary patterns, rather than isolated nutrients, may influence PMS symptomatology through combined metabolic and neuroendocrine effects. An important consideration is the bidirectional relationship between premenstrual symptoms and dietary behaviors, as symptom-related changes in appetite and food preferences may influence dietary intake. From a clinical perspective, nutrition should therefore be considered a complementary strategy rather than a stand-alone treatment, particularly in women with mild to moderate symptoms. Overall, current evidence remains insufficient to support definitive dietary recommendations, underscoring the need for well-designed prospective and interventional studies.

### 4.2. Comparison with the Existing Literature and Study Limitations

The literature on premenstrual syndrome is characterized by marked heterogeneity in prevalence estimates and associated factors, including nutritional variables. This variability is largely attributable to methodological inconsistency, particularly the use of different diagnostic criteria (ACOG, DSM, ICD, ISPMD) and the frequent reliance on retrospective symptom assessment, which may lead to symptom overestimation and limit comparability across studies [6,84]. These limitations are especially evident in studies examining dietary factors in PMS. Taken together, these limitations underscore the need for standardized diagnostic approaches and culturally sensitive methodologies in future epidemiological research. Most available evidence derives from cross-sectional or observational designs, precluding causal inference, while interventional studies are often constrained by small sample sizes, short durations, heterogeneous dietary exposures, and non-standardized outcome measures [6,107]. Moreover, many investigations focus on individual nutrients rather than overall dietary patterns and inadequately control for confounding factors such as body composition, physical activity, stress, lifestyle behaviors, and psychiatric comorbidities [84,108]. Despite these constraints, recent reviews suggest that certain dietary patterns may be associated with improvements in selected PMS symptom domains, particularly affective symptoms. Diets rich in unprocessed foods appear linked to lower symptom severity, whereas Western-style dietary patterns are more frequently associated with worse symptom profiles [84,108]. However, current evidence remains insufficient to support definitive dietary recommendations [6,107]. Overall, these findings highlight the need for well-designed prospective studies using standardized diagnostic criteria and integrated assessments of diet, biological, and psychosocial factors to clarify the role of nutrition in PMS management.

## 5. Conclusions

Premenstrual syndrome in its various forms is a chronic hormone-related disorder that can reduce women’s quality of life. Drug therapy and psychological support are certainly essential in reducing symptoms. At the same time, drug therapies can be ineffective or cause many side effects. Complementary therapy, and specifically nutrition, can play a synergistic role in treating symptoms and improving the overall health of those affected. In this context, general food-based nutritional strategies—rather than structured or prescriptive dietary plans—may be considered as supportive measures, aimed at promoting a balanced intake of nutrients while minimizing symptom-related stress associated with restrictive dietary choices.

In some cases, especially when minor symptoms are present in women seeking to become pregnant, nutrition may even be the only therapeutic strategy, as it does not affect fertility. From a practical perspective, dietary patterns characterized by a higher consumption of fruits, vegetables, whole grains, legumes, fish, and other sources of unsaturated fats, together with a reduced intake of red and processed meat and saturated fats, may represent feasible complementary approaches. Finally, an adequate diet can also have benefits for the comorbidities often associated with premenstrual syndrome. These considerations further support the role of nutrition as a complementary, non-pharmacological strategy that may contribute to symptom relief and overall well-being without replacing established medical treatments.

## Figures and Tables

**Table 1:** Main symptom domains in premenstrual syndrome.

Domain	Common Symptoms	Clinical Considerations
**Affective/emotional**	Irritability, anger, emotional lability, depressed mood, anxiety	Often predominant and more disabling in PMDD
**Cognitive**	Poor concentration, reduced interest, loss of control	Overlap with anxiety/depression; cyclicity is crucial
**Behavioral**	Sleep disturbances, food cravings, hyperphagia, social withdrawal	May mimic primary mood disorders without cycle documentation
**Somatic**	Mastalgia, bloating, headache, myalgia, bowel habit changes	Highly visible symptoms, insufficient alone without functional impact

**Table 2:** Main classification systems for premenstrual conditions.

System	Scope	Symptom Threshold	Key Requirements	Limitations
**ACOG**	Gynecology	≥1 affective or somatic symptom	Functional interference, cyclicity, often prospective confirmation	Less specific for PMDD
**DSM-5-TR**	Psychiatry	≥5 symptoms	At least one mood symptom, defined time window, exclusion of PME	Risk of underdiagnosing disabling subthreshold cases
**ISPMD**	Multidisciplinary	No fixed threshold	Centrality of cyclicity and functional impact	Less standardized for epidemiology
**ICD-10**	Clinical/epidemiological	Variable	Frequently used in observational studies	Limited overlap with DSM criteria

**Table 3:** Macronutrients and effects on PMS.

Author (Year)	Article Title	Study Design	Level of Evidence	Effects on PMS
Houghton et al. (2018) [74]	Carbohydrate and fiber intake and the risk of premenstrual syndrome	Prospective cohort study	High	No association with fiber, carbohydrate, or protein intake
Hashim et al. (2019) [75]	Premenstrual Syndrome Is Associated with Dietary and Lifestyle Behaviors among University Students	Cross-sectional study	Moderate	Higher fat and simple carbohydrate intake; lower protein intake
Cross et al. (2001) [76]	Changes in nutrient intake during the menstrual cycle of overweight women with PMS	Observational longitudinal study	Moderate	Inverse association with fish and seafood intake
Freeman et al. (2002) [77]	Treatment of premenstrual syndrome with a carbohydrate-rich beverage	Nutritional intervention study	Moderate	Carbohydrate intake modulated PMS symptoms
Taheri et al. (2023) [78]	Dietary intake of micronutrients are predictor of PMS	Observational predictive study	Moderate	Simple carbohydrates and fried foods associated with worse symptoms
MoradiFili et al. (2020) [79]	Dietary patterns are associated with premenstrual syndrome	Case–control study	Moderate	Western diet increases risk; healthy patterns protective
Asarian & Geary (2007) [80]	Estradiol enhances lipid-induced satiation	Animal experimental study	Low	Indirect mechanistic evidence only
Farasati et al. (2015) [81]	Western dietary pattern is related to PMS	Case–control study	Moderate	Fruit intake reduces psychological symptoms
Houghton et al. (2017) [82]	Dietary fat and fat subtypes and PMS risk	Prospective cohort study	High	Stearic acid protective; maltose increases risk
Houghton et al. (2019) [83]	Protein intake and the risk of PMS	Prospective cohort study	High	No association with protein intake
Oboza et al. (2024) [84]	Relationships between PMS and Diet Composition	Narrative review	Moderate	No association with protein intake

**Table 4:** Micronutrients, Diet and Complementary Treatments in PMS.

Author (Year)	Article Title	Study Design	Level of Evidence	Main Effects on PMS
Oboza et al., 2024 [84]	Relationships between PMS and Diet Composition, Dietary Patterns and Eating Behaviors	Cross-sectional observational study	Low	Lower intake of calcium, magnesium and potassium associated with PMS
Chocano-Bedoya et al., 2013 [85]	Intake of selected minerals and risk of premenstrual syndrome	Prospective cohort study	Moderate	Calcium intake associated with reduced risk and severity of PMS
Abdi et al., 2019 [86]	Role of vitamin D and calcium in premenstrual syndrome	Systematic review	High	Vitamin D and calcium deficiency associated with PMS
Bertone-Johnson et al., 2010 [87]	Dietary vitamin D intake, 25-hydroxyvitamin D3 levels and PMS	Observational study	Low	Low vitamin D levels associated with increased symptom severity
Rajaei et al., 2016 [88]	Serum vitamin D level and PMS in Iranian women	Case–control study	Low	No significant association between vitamin D and PMS
Bahrami et al., 2018 [89]	High-dose vitamin D supplementation in adolescents	Clinical trial	Moderate	Reduced dysmenorrhea and PMS symptoms
Tartagni et al., 2016 [90]	Vitamin D supplementation for PMS-related mood disorders	Randomized controlled trial	High	Improved mood symptoms and dysmenorrhea
Karimi et al., 2018 [91]	Calcium plus vitamin D in PMS treatment	Randomized controlled trial	High	Improved mood-related PMS symptoms
Arab et al., 2019 [94]	Vitamin D and PMS: systematic review and meta-analysis	Systematic review and meta-analysis	Very high	Reduced severity of PMS symptoms
Zeitoun et al., 2021 [95]	Genetics of iron metabolism and premenstrual symptoms	Mendelian randomization study	Moderate	Higher non-heme iron intake associated with lower PMS risk
Jafari et al., 2020 [96]	Effect of zinc supplementation on PMS	Double-blind randomized controlled trial	High	Reduced physical and psychological symptoms; improved quality of life
Chocano-Bedoya et al., 2011 [97]	Dietary B vitamin intake and incident PMS	Prospective cohort study	Moderate	Higher B vitamin intake associated with lower PMS risk
Soheila et al., 2016 [98]	Effects of vitamin B6 on PMS	Systematic review and meta-analysis	High	Overall symptom improvement, heterogeneous results
Abdollahifard et al., 2014 [99]	Effects of vitamin B1 on PMS symptoms	Meta-analysis	High	Improvement of physical and psychological symptoms
Samieipour et al., 2016 [100]	Effect of calcium and vitamin B1 on PMS severity	Randomized controlled trial	High	Significant reduction in symptom severity
Mohammadi et al., 2022 [101]	Effect of omega-3 fatty acids on PMS	Systematic review and meta-analysis	High	Reduction in PMS symptoms; duration-dependent
Rossignol et al., 1990–1991 [102,103]	Caffeine-containing beverages and PMS	Observational studies	Low	Higher caffeine intake associated with more severe symptoms
Caan et al., 1993; Purdue-Smithe et al., 2016 [104,105]	Caffeine/coffee intake and PMS	Observational study and prospective cohort	Low–Moderate	No consistent association with PMS
Höller et al., 2024 [106]	Use of Vitex agnus-castus in menstrual cycle disorders	Retrospective longitudinal cohort study	Moderate	Improved dysmenorrhea, mastodynia and quality of life

## Data Availability

The data that support the findings of this study are available from the corresponding author, E.P, upon reasonable request.
